# Development of the Japanese version of the general practice assessment questionnaire: measurement of patient experience and testing of data quality

**DOI:** 10.1186/s12875-018-0873-8

**Published:** 2018-11-28

**Authors:** Tsunetaka Kijima, Kenju Akai, Akira Matsushita, Tsuyoshi Hamano, Keiichi Onoda, Shozo Yano, Toru Nabika, Yutaka Ishibashi, Shunichi Kumakura

**Affiliations:** 1 Department of General Medicine, Faculty of Medicine, Shimane University, 89-1, Enya-cho, Izumo-shi, Shimane 693-8501 Japan; 2 Centre for Community-based Healthcare Research and Education, Shimane University, 89-1, Enya-cho, Izumo-shi, Shimane 693-8501 Japan; 3 Nagi Family Clinic, Tsuyama Family Clinic, Yunogou Family Clinic, Family Practice Centre of Okayama, 292-1, Toyosawa, Nagi-cho, Katsuta-gun, Okayama, 708-1323 Japan; 4 Department of Sports Sociology and Health Sciences, Faculty of Sociology, Kyoto Sangyo University, Kamigamomotoyama, Kita-ku, Kyoto-shi, Kyoto, 603-8555 Japan; 5 Department of Neurology, Faculty of Medicine, Shimane University, 89-1, Enya-cho, Izumo-shi, Shimane 693-8501 Japan; 6 Department of Laboratory Medicine, Shimane University, 89-1, Enya-cho, Izumo-shi, Shimane 693-8501 Japan; 7 Department of Functional Pathology, Faculty of Medicine, Shimane University, 89-1, Enya-cho, Izumo-shi, Shimane 693-8501 Japan; 8 Department of Medical Education and Research, Faculty of Medicine, Shimane University, 89-1, Enya-cho, Izumo-shi, Shimane 693-8501 Japan

**Keywords:** General practice assessment questionnaire, Primary care, Physician-patient communication, Interpersonal performance, Quality

## Abstract

**Background:**

Physicians’ interpersonal performance is critical in medical practice, especially primary care practice. The General Practice Assessment Questionnaire (GPAQ) was developed in the United Kingdom to evaluate the quality of primary care from the viewpoint of patients. This questionnaire highlights the evaluation of interpersonal skills and interactions between physicians and patients. Though several other tools also exist to evaluate primary care quality, the GPAQ has several distinctive evaluation items, covering receptionists, access to primary care, and enablement (patients’ understanding of self-care and of their own health after consultation). Our purpose was to develop and validate a Japanese version of the GPAQ.

**Methods:**

This cross-sectional study tested the validity and reliability of the Japanese version of the questionnaire. We translated the original GPAQ into Japanese and assessed its reliability and validity among patients aged ≥20 years at five rural primary care centres located in Shimane and Okayama prefectures, Japan. We also examined its internal reliability using Cronbach’s alpha coefficient and construct validity—including item-scale correlations, item-other scale correlations, and inter-scale correlations. Moreover, we examined correlations between each score and overall satisfaction using Spearman’s correlation coefficient for criterion-related validity.

**Results:**

The translated version of the GPAQ was administered, and we received 252 responses (mean age: 68 ± 12.3 years, male: 42.9%); all data were analysed. The translated questionnaire showed good reliability and validity, with Cronbach’s alphas ranging from 0.79–0.92 for all scales, and satisfactory item-scale, item-other scale, and inter-scale correlations. Correlations with overall satisfaction were strong (Spearman’s correlation coefficients: 0.31–0.38) for all scales except ‘continuity of care’.

**Conclusions:**

The Japanese version of the GPAQ was acceptable, reliable, and valid. This could be a useful instrument to evaluate key areas of primary care performance in Japan, particularly physicians’ communication skills. Further work is required to evaluate its utility in urban areas.

**Electronic supplementary material:**

The online version of this article (10.1186/s12875-018-0873-8) contains supplementary material, which is available to authorized users.

## Background

Improving the quality of medical care is a high priority in Japan, as well as in other countries. There are many tools worldwide for evaluating the quality of primary care, including the Components of Primary Care Index [1], Primary Care Assessment Survey (PCAS) [2, 3], Primary Care Assessment Tool (PCAT) [4], EUROPEP questionnaire [5], General Practice Assessment Survey (GPAS) [6, 7], and General Practice Assessment Questionnaire (GPAQ) [8]. Among these tools, the GPAQ, was originally developed in the United States as the PCAS; then, in collaboration with the Health Institute in Boston and the National Primary Care Research and Development Centre at the University of Manchester, in the United Kingdom, it was modified for use in British general practice and became the GPAS, which has been widely used [3, 6, 7, 9]. A brief version of the GPAS (the GPAQ) was also used for increased accessibility to patients and with a revised scoring system [8]. The GPAQ has been broadly used as a quality survey for general practice and has been translated into and validated in many languages, including Chinese, Somali, and Thai [10–12]. The goal of this questionnaire is to evaluate various aspects of care quality, such as receptionists’ demeanour, access to primary care, continuity of care, physicians’ communication skills, and enablement (understanding of self-care and of one’s own health after consultation).

It has been noted that effective communication skills among physicians are critical for patients to develop trust in them [13]. Factors such as maintaining confidentiality, respecting patients’ dignity, and involving them in treatment decisions are also highly valued [14]. In addition, systematic patient feedback about physicians’ interpersonal skills has been shown to help improve physicians’ interpersonal performance [14, 15]. Thus, the GPAQ is used to assess how patients feel about their primary care and how they cope with their problems or illnesses during and after consultations, making it an enablement scale [16].

In Japan, however, no study has used the GPAQ in primary care; instead, a Japanese version of the PCAT (JPCAT) has been developed and used [17]. Previous studies using the JPCAT have reported that patients’ experience in primary care affects their health-associated behaviour or ability in Japan. Patient experience of primary care was positively related to breast cancer screening uptake in Japanese women [18], while longitudinality and comprehensiveness of primary care service were related to higher health literacy [19]. On the other hand, Japanese family physicians (FPs) and general practitioners (GPs) require additional education. An Organisation for Economic Co-operation and Development (OECD) review noted that Japanese primary care has been provided by a cadre of semi-generalists/semi-specialists, those who were working as generalists in the community with an unspecified amount of time to train as generalists (no compulsory further training) after they had left hospital practice [20]. Therefore, the former Japanese Academy of Family Medicine (JAFM) (currently the Japan Primary Care Association (JPCA)) started the certification system of a residency training programme for FPs in 2006 [21]. Moreover, The Japanese Medical Specialty Board established primary care as a distinct new medical specialty beginning in 2018 [22]. Patients in European countries are generally provided consultation by GPs who completed specialist training in family medicine, and some of those countries have a system for registering patients for primary care [23]. A registration system with a primary care practitioner is suggested to possibly bring more effective primary care such as providing lifestyle advise, coordinating an individual’s care, and strengthening the relationship between patients and their primary care physicians [23]. There is no such registration system in Japan; however, Japan’s medical system is gradually beginning to recognise primary care physicians as medical specialists. For example the Japanese Medical Specialty Board recognised family medicine as a new specialty in 2017 [22]. The GPAQ is a tool to assess physicians’ interpersonal skills; thus, we expected its results to be useful not only for improving the quality of care but for educating new FPs/GPs.

Compared with the PCAT, the GPAQ features a more detailed evaluation of practitioners’ interpersonal and communication skills in relation to patients. To take advantage of this affordance, we focused on the development and validation of a Japanese version of the tool (GPAQ-J). To verify the robustness of the GPAQ-J, we also referred to the results of the JPCAT, an established primary care assessment tool.

Thus, the purpose of the current study was to develop the GPAQ-J, a self-administered questionnaire, from the original GPAQ, using a formatted translation process, and to conduct a full validation survey. We established the utility of this new tool through statistical analyses. We also analysed responses to questionnaire items on physicians’ interpersonal skills in their practice. The results elucidate the characteristics of Japanese primary care and show the differences between this new tool and an existing alternative—the JPCAT—for the Japanese context.

## Methods

### Design and development of the GPAQ-J

First, we conducted a cross-sectional study to develop the items of the GPAQ-J; then we examined the validity and reliability of this questionnaire. Next, we compared the GPAQ-J with the JPCAT. The process included seven steps, following previous studies [10, 17, 24]. To capture cultural differences, those studies conducted qualitative as well as quantitative assessments [10, 17, 24]. Qualitative methods improve the contents of a questionnaire using comments offered by non-expert and expert panels. We modified sentences or added items using participants’ ideas and adapted or eliminated items based on quantitative assessment, which incorporated focus groups and the Delphi method. The Delphi method assesses quantitative inference using qualitative methods and was used to review the expert opinions. In the original Delphi method, the process creates a list to review such as a detailed literature review or a list of diagnostic criteria [25]; however, we started by reviewing the translated questionnaire without the formal process of creating a list; hence, our process used a modified Delphi method. The procedure was as follows: 1) researchers selected appropriate participants and sent them the questionnaire; 2) participants evaluated the appropriateness of the questionnaire and provided comments; 3) researchers judged participants’ responses and modified the items accordingly; 4) researchers sent participants the results of the assessment and the revised questionnaire; and 5) participants assessed the revised questionnaire and offered comments again. This procedure was repeated until a consensus was reached. A focus group was also conducted to review and improve the instrument’s content. For qualitative analysis, in Steps 2 and 3, two analysts—a primary care physician and a health care research expert—analysed the results.

*Step 1. Forward translation:* Two native Japanese professional Japanese–English translators independently translated the GPAQ into Japanese. Each had clinical experience in a Japanese medical field and more than eight years of translation experience. Subsequently, another expert translator synthesized the two versions into one.

*Step 2. Qualitative review (medical expert panel):* The developers of the GPAQ-J, who were primary care experts, examined its clarity, accuracy, and adequacy by conducting a review using a modified Delphi method [25]. The developers were 12 primary care experts from four professions (three from each profession): primary care physicians who were JPCA-certified FPs, community-health centre directors, healthcare research experts, and experienced primary care nurses who were trained in family medicine or experienced family-medicine nurse supervisors. They evaluated the necessity of each item using a nine-point scale and were also asked about necessary revisions and additions. The consensus was judged based on a rating score; a perfect or very good score implied consensus [26]. ‘Perfect’ referred to when all responses ranged from 7 to 9. ‘Very good’ meant that 80% of the responses ranged from 7 to 9 and 80% were within two integers of the median. Participants’ comments were also used to revise, remove, or add items. Three rounds were conducted until a consensus was reached.

*Step 3. Qualitative review (non-medical panel):* A focus group was conducted with 10 participants from outside the medical profession to ensure that the questionnaire was easy to understand and used common language. The participants were instructed to check whether items were culturally valid and useful, flag troublesome items, and propose alternatives.

*Step 4. Back translation:* Following previous studies [17, 24], a back translation of the GPAQ-J was conducted by a native-English-speaking writer and translator who had lived in Japan for more than 10 years.

*Step 5. Expert review:* The health research experts and the forward and back translators jointly reviewed all items, discussed whether they were appropriately translated, and raised any concerns. Finally, the questionnaire was adjusted to the most appropriate items, which participants had agreed upon through the expert review.

*Step 6. Pilot testing:* A pilot test was conducted with 35 patients in three local primary care centres in Japan; then, final revisions were performed.

*Step 7. Full validation study:* Participants included Japanese patients aged ≥20 years who had ever undergone consultation at a primary care centre. A research assistant informed participants of the purpose and content of the study with an explanation form. A returned, completed questionnaire was considered consent to participate in this study. Japanese patients were defined as those living in Japan who could adequately understand Japanese. Japanese primary care physicians’ training differs from those in other OECD countries [20]; therefore, it took time to establish a FP/GP training system in Japan. Until JAFM started the residency training programme for FPs in 2006 [21], there was no standardised training system for primary care physicians in Japan. Patients’ experiences largely depended on the individual physician’s career. For the validation study, we targeted respondents with similar patient experiences to minimize variation in the data; the experience of patients seeing physicians who had just left hospital practice would differ from that of patients seeing physicians trained in family medicine. Hence, we focused on the primary care centres where physicians who had been educated or trained in family medicine provided primary care. For this study we defined a primary care centre as a facility specialising in family medicine practice with the qualifications for residency training; specifically, the practice had to have one or more certified FPs holding a diploma in primary care from the JPCA. A previous study also noted that patients’ experience or satisfaction depended on the location of the healthcare facility—in particular whether it was in a rural or an urban area [27]. Therefore, we collected data by restricting ourselves to experiences in rural primary care centres. A rural area was defined as a region with a population density lower than the national average in Japan (approximately 343 people per km^2^) [28]. Finally, we selected five primary care centres located in Shimane and Okayama prefectures where the mean population density was approximately 122 people per km^2^.

Ultimately, the GPAQ-J contained six sets of questions to assess the quality of primary care: frequency of visits (item 1), helpfulness of receptionists (item 2), access to care (items 3–5), continuity of care (item 6), doctors’ communication skills (item 7), enablement (item 8), demographic data (items 9–12), and general comments (item 13). Demographic data gathered included age, sex, employment, and health status. Overall satisfaction with primary care (1 = *very dissatisfied*, 3 = *neutral*, and 5 = *very satisfied*) was also assessed.

We had concerns about the evaluation of patient satisfaction using the GPAQ-J, because it was self-administered, completed at the health centre, contained patients’ demographic information, and included sensitive questions about their physicians’ communication skills which they responded to just after the consultation. Therefore, the JPCAT was also administered at the same medical facilities during the same period. However, to reduce the burden (particularly for the elderly), this instrument was given to some of the same sample of patients as the GPAQ-J. On the JPCAT, patients’ demographic data were not recorded, because the original questionnaire did not include those data. Additionally, we intended to compare patients’ satisfaction ratings between the two scales to confirm whether patients had overestimated their satisfaction on the GPAQ-J; previous studies have found that demographic questions can affect survey measurements and that sensitive data are likely to be misreported when demographic data are also collected [29, 30]. Respondents were given small gifts of gratitude worth JPY 300 (approximately 3 USD). This study was approved by the Shimane University Institutional Committee on Ethics (approval number: 2763).

### Statistical analyses

First, we assessed each item and defined missing data as out-of-range responses, missing values, and ‘does not apply’ values. Each item was assessed to determine the percentage of responses falling into the highest and lowest categories (i.e. ‘very poor’ and ‘excellent’). Meaningful ceiling and floor effects were defined as more than 40% of responses, following a previous study [17]. Second, we calculated scaled scores for the GPAQ scales using manual procedures, with zero as the lowest possible score and 100 as the highest possible score [11].

Third, we calculated internal reliability, construct validity (including convergent validity and discriminant validity), and criterion-related validity for the GPAQ-J. Cronbach’s alpha of 0.70 was set as the lowest acceptable value for internal reliability [31, 32]. Convergent validity was tested by confirming whether each item was correlated with the hypothesised scale; we defined item-scale correlations above 0.3 as acceptable [6, 17, 33, 34]. Equal item-scale correlations were assessed by calculating the range of correlations obtained for all items in a scale, and we defined an acceptable narrow range as < 0.2 [32, 33]. Discriminant validity was assessed using item-other scales correlations. If the correlation between an item and the hypothesised scale was significantly greater than the correlations between the item and all the other scales, item-discriminant validity was confirmed [35, 36]. A scaling success rate was also calculated [32, 37]. Inter-scale correlations were used to assess construct validity [6, 38] and evaluate how distinct the items between and within scales are; if inter-scale correlations are lower than internal reliability measures (Cronbach’s alpha), each scale measures a unique concept [39, 40]. Moreover, we examined correlations between each scale/item and overall satisfaction using Spearman’s correlation coefficient for criterion-related validity, and defined correlations > 0.30 as meaningful [41].

Fourth, we assessed the dimensional structure of the GPAQ-J using principal component analysis. The aim was to explore a smaller number of hypothetical factors within multiple observed factors. We initially identified the number of factors by examining a scree plot. Next, we performed a full principal axis factor analysis based on this number of factors. Furthermore, a varimax rotation procedure was conducted to produce the solutions and uncorrelated factors. This procedure is similar to previous factor analyses of the original GPAS and GPAQ [8, 9].

Finally, we analysed the collected data following the JPCAT method [17, 42]. We assessed overall satisfaction and its correlation with each scale of the JPCAT to determine the external validity of the GPAQ-J. All statistical analyses were carried out using JMP Pro 12 (SAS Institute Inc., Cary, NC, USA).

## Results

We developed the GPAQ-J through the seven steps described above. Patients (*N* = 252) responded to the GPAQ-J (approximately 50 participants per clinic). Respondents were Japanese patients who had visited any of the five target primary care centres from May to July 2017. These respondents accounted for approximately 6.7 ± 2.1% of outpatients aged ≥20 years in each clinic. Patients (*N* = 234) also responded to the JPCAT at the same clinics during the same period; however, 19 did not identify a primary care provider. Therefore, 215 responses were included in the final analyses.

The process of the expert review and pilot test are in Additional file 1. The item ‘Waiting time at the practice for each consultation’ required revision in Steps 2 and 3. In addition, items about patients wanting to see a particular physician/any physician and about urgent consultations were removed in Step 2. Additionally, we added a question in Step 2, and revised it in Steps 3 and 4, about telephone management by physicians and nurses; because, in Japan, when patients telephone for medical advice, it is often nurses who handle the call. In Step 2, for cultural reasons, questions about ethnicity were removed, as there are few sizable ethnic groups in Japan. Ultimately, the GPAQ-J was finalized (see Additional file 2, content in Japanese). The main procedure of this study was not modified during the research.

Across all clinics, 18 physicians (5.1 ± 2.4 physicians per clinic) were evaluated for consultation skills. We found no floor or ceiling effects for the GPAQ-J scales; mild ceiling effects were found for some individual items on the GPAQ-J, and moderate ones on the JPCAT (see Additional files 3 and 4). Three-fourths of patients reported having seen a physician seven or more times in the previous 12 months. Regarding physicians’ communication skills, 84% of patients responded to the item ‘How well the physician listened to what patients had to say’ with ‘very well’ (for other items see Additional file 3).

Table 1 shows respondents’ demographic characteristics. All respondents who agreed to participate answered the questionnaire.

**Table 1:** Patients’ characteristics (*N* = 252)

Characteristic	Number	%
Age (years)		
Mean ± SD	68.3 ± 12.3	
Range (years)	28–95	
n	230	
Sex		
Male	99	
Female	132	
n	231	
Number of primary care visits		
Once/year	1	0.4
2–6 times/year	59	24.5
7–12 times/year	121	50.2
13 or more times/year	60	24.9
n	241	
Chronic illness^a^		
Yes	134	68.7
No	61	31.3
n	195	
Occupation		
Employee/public servant	28	12.6
Employer	33	14.9
Student	0	0
Part-time worker	30	13.5
Unemployed	102	45.9
Other	29	13.1
n	222	

*SD* standard deviation

^a^Chronic illness means any long-term health problem (illness or disability), which can negatively impact all aspects of a person’s life

Table 2 shows the central tendency, dispersion, and mean scores for all scales and patients’ overall satisfaction with the GPAQ-J.

**Table 2:** General Practice Assessment Score in five primary care centres using the GPAQ-J (*N* = 252)

Scale	Number of items	Mean score	SD	25th percentile	50th percentile	75th percentile	Floor%	Ceiling%	Range of scores
Receptionists	1	74.9	20.1	60	80	80	1.6	20.9	0–100
Access	5	64.9	16.1	55	66.7	76	0	3.7	20–100
Continuity of care	1	67.5	21.9	60	80	80	1.0	13.6	0–100
Communication	8	81.6	13.4	74.5	80	95	0	15.6	45–100
Enablement	2	65.8	22.7	50	66.6	83.3	0	19.5	33.3–100

*SD* standard deviation, *GPAQ-J* Japanese version of General Practice Assessment Questionnaire

Table 3 shows the results of validity and reliability testing of the GPAQ-J. All item-scale correlations exceeded 0.6, and all scales demonstrated a narrow range of item-scale correlations. The reliability test was implemented to confirm item consistency for access to care, communication, and enablement. Cronbach’s alpha for all scales was > 0.70. Inter-scale correlations were lower than each value of Cronbach’s alpha. Item-other scales correlations were significantly lower than item-own hypothesised scale correlations for access, communication, and enablement (detailed statistics in Additional file 5). The scaling success rate was 100% for all scales. Moreover, all scales except continuity of care were significantly correlated with overall satisfaction using Spearman’s correlation coefficient (*p* < .001).

**Table 3:** Results of validity testing and internal consistency from GPAQ-J (*N* = 252)

Scale	Number of items	Item-scale correlations	Item-other scales correlations	Scaling success rate^a^ (%)	Inter-scale correlations	Cronbach’s alpha	Correlations with overall satisfaction^b^
Receptionists	1	NA	0.26–0.53	NA	0.26–0.54	NA	0.38
Access	5	0.66–0.81	0.15–0.51	25/25 (100)	0.37–0.54	0.79	0.38
Continuity of care	1	NA	0.28–0.45	NA	0.28–0.45	NA	0.17
Communication	8	0.77–0.87	0.23–0.45	40/40 (100)	0.36–0.51	0.92	0.37
Enablement	2	0.92–0.93	0.24–0.41	10/10 (100)	0.26–0.41	0.83	0.31
Total	17					0.9	

*NA* not applicable because there is only one item in those key areas, *GPAQ-J* Japanese version of General Practice Assessment Questionnaire

^a^The scaling success rate was calculated as follows: the denominator is the total number of item-scale correlations tested, and the numerator is the number of those correlations in which the items in the scale correlate significantly greater with their own hypothesised scale than with other scales

^b^Spearman’s correlation coefficient

Table 4 shows the results of validity and internal reliability testing of the JPCAT. Cronbach’s alpha was > 0.70 for all scales, item-scale correlations were > 0.3 for all scales, and item-other scales correlations were significantly lower than item-own hypothesised scale correlations, except for the community orientation scale. Longitudinality, comprehensiveness (services available), and community orientation were significantly correlated with overall satisfaction.

**Table 4:** Results of validity testing and internal consistency from JPCAT (*N* = 215)

Scale	Number of items	Item–scale correlations	Item–other scales correlations	Cronbach’s alpha	Correlations with overall satisfaction^a^
First contact (Access)	3	0.62–0.92	0.009–0.34	0.78	0.22
Longitudinality	5	0.74–0.88	0.09–0.57	0.86	0.38
Coordination	6	0.73–0.86	0.09–0.52	0.89	0.23
Comprehensiveness (services available)	5	0.81–0.89	0.03–0.57	0.91	0.40
Comprehensiveness (services provided)	5	0.58–0.82	−0.04–0.40	0.81	0.26
Community orientation	5	0.50–0.81	−0.11–0.45	0.78	0.44
Total	28			0.90	0.47

*JPCAT* Japanese version of the Primary Care Assessment Tool. ^a^Spearman’s correlation

The mean score for overall satisfaction was 4.2 ± 0.9 on the GPAQ-J and 4.4 ± 0.6 on the JPCAT. Thus, satisfaction with the GPAQ-J was lower (*p =* .03 by Wilcoxon rank-sum test; see Additional files 3 and 4)

Table 5 shows the principal component analysis using a rotated factor matrix. Analysis of the scree plot identified three factors, which accounted for 62.4% of the variance. Item loadings > 0.3 on a factor are considered substantive and are shown in bold in Table 5. Component 1 included only the communication scale, which explained 31.1%. Component 2 included the receptionists scale, access scale, and continuity of care scale, which explained 20.7%. Component 3 included only the enablement scale, which explained 10.6%. In component 2, the continuity of care scale was weakly correlated with access (Spearman’s correlation coefficient: 0.29); the access scale was strongly correlated with the receptionists scale (Spearman’s correlation coefficient: 0.53).

**Table 5:** Rotated factor matrix of the analysis of the GPAQ-J

No.	Item	Component 1	Component 2	Component 3
2	Receptionists	0.179	**0.633**	0.081
3a	Availability of surgery (any GP)	0.247	**0.529**	−0.008
4b	Waiting times at surgery	0.108	**0.665**	−0.044
5a	Quality of phone call (length of time, etc.)	0.137	**0.775**	0.175
5b	Availability of doctors on the phone	0.245	**0.702**	0.245
5c	Doctor’s or nurse’s phone support	0.139	**0.795**	0.127
6b	Continuity of care	0.223	**0.495**	0.136
7a	GP questioning	**0.706**	0.202	0.121
7b	GP attention	**0.832**	0.167	0.109
7c	GP putting you at ease	**0.817**	0.291	0.032
7d	GP involving you in decisions	**0.84**	0.188	0.179
7e	GP explanations	**0.794**	0.194	0.209
7f	GP spending time with you	**0.631**	0.263	0.078
7 g	GP patience	**0.848**	0.198	0.134
7 h	GP caring and concern	**0.779**	0.154	0.152
8a	Able to understand your problem	0.266	0.196	**0.857**
8b	Able to keep yourself healthy	0.192	0.15	**0.885**

*GP* general practitioner, *GPAQ-J* Japanese version of General Practice Assessment Questionnaire

Items loading >0.3 on a component are considered substantive, and those are bolded

Table 6 shows inter-country comparisons between our data and GPAQ results for Thailand, New Zealand, Australia, and the UK [10, 43, 44]. There are a few differences in content and in GPAQ format across the studies, such as a consultation version (administered in the primary care facility) or postal version (administered by mail). Results showed that mean scores from our study were higher than Thailand and the UK, and lower than New Zealand and Australia.

**Table 6:** Inter-country comparison (mean score)

	Our data (Japan)	Thailand	New Zealand	Australia	UK
Receptionists	74.9	63.5	85.7	81.8	69.1
Access	64.9	55.1	76.6	68.6	58.3
Continuity of care	67.5	65.8	80.4	76.5	66.1
Communication	81.6	69.0	89.1	84	75.9
Enablement	65.8	80.8	NA	NA	NA
Overall satisfaction	80.4	80.6	86.3	81.6	76.5

*NA* not applicable, *UK* United Kingdom

Moreover, we examined the correlations between each item and overall satisfaction. In the GPAQ-J, items ‘Does the doctor care and show concern for you?’ ‘How about the service of the receptionist?’ ‘Did the doctor explain your problems and treatment sufficiently?’ and ‘Did the doctor listen to your questions or worries with patience?’ were significantly correlated with overall satisfaction (Spearman’s correlation coefficients were 0.40, 0.38, 0.37, and 0.36, respectively; all ps < .001).

## Discussion

The GPAQ-J demonstrated good reliability and validity, which confirms that it is an acceptable instrument to evaluate primary care in Japan. We analysed the data based on the original version of the GPAQ, and we did not amend the main scales. Based on the original scale classification, we analysed reliability and validity, and applied principal component analysis. Since the GPAQ has been widely used since 2004 [8, 11, 45], we did not modify its basic structure. In addition, during the development of other versions of the GPAQ, factor analysis was not conducted as part of the initial analysis [8, 10]. Therefore, we conducted factor analysis to confirm whether those categories of GPAQ-J such as receptionists, access, continuity of care, communication, and enablement were compatible with our data to reconfirm robustness.

As for the researchers’ role, two researchers were included in the expert review in Step 5. One author was not a principal researcher but took part in the qualitative review in Step 2. The principal researcher contacted all members and compiled their comments in Step 2. One of the authors worked at one of the primary care centres, and another had previously worked there. To avoid participant or experimenter bias, no researcher was a research assistant. The principal researchers analysed all data, including detailed factor analysis, which might cause potential bias. However, that approach was taken based on previous GPAQ studies [8, 11].

Almost all researchers had worked in rural areas, and thus recognized the need to evaluate the quality of primary care in rural areas. The proportion of elderly people in rural areas in Japan is increasing, so the results might potentially be particularly important for eldercare—a growing concern in Japan as the population ages. The study population was mainly elderly people, which affected participant recruitment. In fact, the elderly population in the areas we surveyed was higher than the overall Japanese elderly population (32.7% vs. 25.1%) [46, 47].

For selection of respondents, staff who were currently working or had previously worked in each facility selected patients they knew in that facility to complete the questionnaire. Since staff selected patients, selection bias is a possible limitation. Subsequently, research assistants explained that collected questionnaires would be managed and analysed outside the facility, to maintain confidentiality; they also conducted the informed consent process. Questionnaires were collected by volunteers after being posted by patients into a box placed in the facility. To avoid the possibility that some patients might feel nervous or constrained in their comments because the informed consent sheet included the names of researchers who had worked with some of them before, the questionnaire did not gather any personal information such as age or sex.

The GPAQ-J demonstrated good reliability and validity, which indicates that it is an acceptable instrument to evaluate primary care in Japan. Owing to differences between medical systems, it was necessary to customise several items from the original GPAQ to the GPAQ-J. For instance, in Japan, it is common for patients to visit a primary care facility without making an appointment. Several physician consultations per person have been reported in Japan compared to other countries by the OECD (Japan, 12.7/year; OECD average, 6.9/year) [48]. In Japan, patients can consult a primary care physician on a work day with ease. This can be both positive and negative; on the one hand, it provides access for patients who urgently want to consult a physician; on the other hand, it is likely that there will be a longer wait time in the facility even for patients with scheduled appointments. Therefore, the item ‘Waiting time at the practice for each consultation’ required repeated discussion and revision. Further, items about patients wanting to see a particular physician/any physician or receive urgent consultation were removed because of the above characteristic of the Japanese medical system.

Although we revised some items, the GPAQ-J retained a Cronbach’s alpha of 0.90. Our principal component analysis showed comparable results to previous research in the UK [8]. Regarding convergent validity, item-total correlations were found to be high (> 0.6) for access, physicians’ communication skills, and enablement. Discriminant validity was demonstrated by the scaling assumption, whereby the correlation of an item with its own hypothesised scale was significantly greater than that with other scales.

Table 5 shows the results for the three major components. A previous study indicated the same categories [8], supporting the compatibility of our scale categories with the data. Factor analysis is used to identify externally meaningful dimensions or tendencies by exploring variables with high factor loading [49]. These were categorised into three dimensions: physicians’ communication skills; demeanour of receptionists, access, and continuity of care; and enablement. The Physicians’ communication skills component had the highest contribution rate in the questionnaire, suggesting that the GPAQ-J is adequate to evaluate physicians’ communication performance. Receptionists were included in a composite component with the access and continuity of care scales, similar to a previous study [8]. Access was strongly correlated with the demeanour of receptionists, which implies that receptionists’ service was a more critical component related to access to primary care facilities than was continuity of care with one physician.

The results for the GPAQ-J were similar to those for the JPCAT regarding total Cronbach’s alpha, construct validity, overall satisfaction, and correlations between each scale and patients’ overall satisfaction. As the questionnaires were not completed by the same participants, these results do not directly validate the GPAQ-J; however, the questionnaires were administered to similar respondents (patients in the same primary care centres during the same period), which indicates external validity with the existing tool, the JPCAT—although the GPAQ-J and JPCAT have different items and scales. Therefore, it is difficult to conduct an exact comparison. On the GPAQ-J, receptionists’ demeanour, access, and physicians’ communication skills were positively related to overall satisfaction, as were, on the JPCAT, community orientation, comprehensiveness, and longitudinality. Although the JPCAT involves a comprehensive primary care evaluation, some items about communication, showing a correlation with overall satisfaction, are included only in the GPAQ-J. For the communication scale, Cronbach’s alpha was high, and item-scale correlations were narrow. The GPAQ-J can, therefore, be considered an appropriate and useful tool for evaluating physicians’ communication skills. Potentially, it could also be applied to evaluate communication training of medical students or trainees or to survey Japanese patients’ satisfaction with their physicians’ communication skills.

For inter-country comparison, we compared our data with those from Thailand, New Zealand, Australia, and the UK [10, 43, 44]. Almost every scale in our data was highly rated compared with Thailand and the UK, but lower-rated than New Zealand and Australia. Our sample and data included older patients who were noted to rate highly receptionists, access, continuity of care, and overall satisfaction compared to younger people [43]. Regarding the difference in access between Japan and Thailand, Thailand data were investigated in a hospital; hospital respondents possibly affected the lower access score compared with primary care facility respondents in Japan. UK surveys were conducted by mail (postal version) which may account for the differences compared to the Thailand and UK data. As for Australia and New Zealand, sample size and the history of family medicine in the respective countries may influence differences in factors such as continuity of care.

Our data were mostly within predictions, except on two points. First, the continuity of care scale was less related to overall satisfaction than the research team expected. We discussed above that the continuity of care scale was based only on evaluation of the consultation with a main physician; in contrast, the continuity of care concept involves understanding the whole patient, including detailed medical history, medication, job, and family condition. Although items covering these factors were included in the communication scale, the continuity of care scale needs to be revised to address them. Second, patients’ overall satisfaction was very high. A previous GPAQ study reported that older respondents tended to express greater satisfaction with their care than did younger ones [43]; therefore, the older population may have influenced the high score.

The study did, however, have some limitations. First, we used only a single back translator. Although some previous studies showed appropriate results with one back translator [10, 17, 24], it may nevertheless be insufficient for comparing the original version with our Japanese version. Second, the GPAQ-J was administered to patients in the waiting room, just after consulting a physician. Immediate evaluation is advantageous to elicit fresh assessments without risk of forgetting; however, it could cause respondents to overestimate satisfaction. In our study, however, overall satisfaction on the GPAQ-J, which included demographic data, was lower than on the JPCAT (without demographic data). Although the questionnaires have some differences, this result suggests that the GPAQ-J did not overestimate satisfaction. Third, concerning comparing the JPCAT and GPAQ-J, some patients responded to both questionnaires sequentially on the same day, some patients on different days, and other patients responded to only one questionnaire. In sequential administrations, the time commitment may increase response burden and lower response rate [50]. Fourth, this survey focused on rural areas, and had fewer participants than in previous GPAQ studies [8, 44]. However, factor loadings in the principal component analysis sufficiently identified items in three components, and the coefficients between scales and overall satisfaction also indicated substantial differences; hence, the sample size was sufficient for our statistical analyses. Fifth, the communication skills of only a limited number of physicians were evaluated; thus, it is possible that there was evaluation bias. Finally, this study was limited to a small percentage of patients who visited one of the five targeted family medicine training centres and were selected mainly by staff working at the facility. There may have been biases at play towards respondents who communicated well with staff and had good feelings about the facility; that is, we may have under-sampled patients who were not satisfied. Further research in a variety of areas and facilities, with larger numbers of physicians, and using a postal method are needed to generalise the results to Japanese primary care.

## Conclusions

We developed a Japanese version of the GPAQ and compared it with the JPCAT. The GPAQ-J demonstrated good validity and internal reliability. Physicians’ communication skills, receptionists’ demeanour, and access were strongly related to patient satisfaction. Our findings indicate that the GPAQ-J can provide valuable information to evaluate the quality of primary care in Japan, particularly for rural areas and elderly people. Japan is now experiencing a dramatic increase in its aged population, which is the highest globally [47], and this survey can appropriately assess primary care in Japan’s current circumstances. Future work needs to validate its appropriateness in a range of other contexts within Japan.

## Additional files

Additional file 1: Results of expert review and pilot test of GPAQ-J. Description of the development process. (DOCX 17 kb)

Additional file 2: Contents of GPAQ-J. Question items – Japanese. (DOCX 15 kb)

Additional file 3: Distribution of each item of GPAQ-J. Item descriptive statistics. (DOCX 17 kb)

Additional file 4: Distribution of each item of JPCAT. Item descriptive statistics. (DOCX 15 kb)

Additional file 5: Inter-scale correlations matrix. Reliability coefficients and inter-scale correlations. (DOCX 14 kb)

## Abbreviations

FP: Family physician
GP: General practitioner
GPAQ: General Practice Assessment Questionnaire
GPAQ-J: Japanese version of General Practice Assessment Questionnaire
JAFM: Japanese Academy of Family Medicine
JPCA: Japan Primary Care Association
JPCAT: Japanese version of the Primary Care Assessment Tool
OECD: Organisation for Economic Co-operation and Development
PCAS: Primary Care Assessment Survey
PCAT: Primary Care Assessment Tool
