# Supplementary material for: Development of the Japanese version of the general practice assessment questionnaire: measurement of patient experience and testing of data quality
Source: BMC Fam Pract. 2018 Nov 28;19:181. doi: 10.1186/s12875-018-0873-8 (PMC6264598; doi:10.1186/s12875-018-0873-8)
Supplement: Supplementary file 1 — Results of expert review and pilot test of GPAQ-J. Description of the development process. (DOCX 17 kb) [file 12875_2018_873_MOESM1_ESM.docx]

| Additional file 1. Results of expert review and pilot test of GPAQ-J | | | | | |
| --- | --- | --- | --- | --- | --- |
| Original No. | Original Questionnaire | Original Answer | Developed Questionnaire | Developed Answer | Expert round (median) |
| 1 | In the past 12 months, how many times have you seen a doctor from your practice? | None, once or twice, three or four times, five or six times, and seven times or more |  | 1 time, 2-6 times, 7-12 times, 13 times or more, and Don’t know/Don’t remember | 8 |
| 2 | How do you rate the way you are treated by receptionists at your practice? |  |  |  | 8 |
| 3a | How do you rate the hours that your practice is open for appointments? |  |  |  | 8 |
| 3b | What additional hours would you like the practice to be open? | Early morning, Lunch times, Evenings, Weekends, None, I am satisfied |  | Currently good, Early morning, During Lunch, Evening (until 9:00), Saturday, Sunday /holidays, and Other (please specify) | 8 |
| 4 | Thinking of times when you want to see a particular doctor |  |  |  |  |
| 4a | How quickly do you usually get to see that doctor? |  | Removed |  | 5 |
| 4b | How do you rate this? |  | Removed |  | 5 |
| 5 | Thinking of times when you are willing to see any doctor |  |  |  |  |
| 5a | How quickly do you usually get seen? |  | Removed |  | 5 |
| 5b | How do you rate this? |  | Removed |  | 5 |
| 6 | If you need to see a GP urgently, can you normally get seen on the same day? |  | Removed |  | 6.5 |
| 7a | How long do you usually have to wait at the practice for your consultations to begin? | 5 minutes or less, 6-10 minutes, 11-20 minutes, 21-30 minutes, and more than 30 minutes |  | Less than 15 minutes, 15-30 minutes, 30-60 minutes, 1-2 hours, and more than 2 hours | 8 |
| 7b | How do you rate this? |  |  |  | 8 |
| 8 | Thinking of times you have phoned the practice, how do you rate the following |  |  |  |  |
| 8a | Ability to get through to the practice on the phone? |  | What did you think about the quality of the phone call (length of time, etc.)? |  | 8 |
| 8b | Ability to speak to a doctor on the phone when you have a question or need medical advice? |  |  |  | 8 |
| 8c | New |  | When you had a question or needed medical advice, how was the doctor's or nurse’s phone support? |  |  |
| 9a | In general, how often do you see your usual doctor? | Always, Almost always, A lot of the time, Some of the time, Almost never |  |  | 8 |
| 9b | How do you rate this? |  |  |  | 8 |
| 10 | Thinking about your consultation with the doctor today, how do you rate the following |  |  |  |  |
| 10a | How thoroughly the doctor asked about your symptoms and how you are feeling |  | Did the doctor ask enough questions about your symptoms and concerns? |  | 8 |
| 10b | How well the doctor listened to what you had to say |  | Did the doctor listen well enough to what you wanted to say? |  | 9 |
| 10c | How well the doctor put you at ease during your physical examination |  | During the physical examination, did the doctor show enough concern for your privacy and pain? |  | 8 |
| 10d | How much the doctor involved you in decisions about your care |  | When determining your treatment, did the doctor adequately discuss it? |  | 9 |
| 10e | How well the doctor explained your problems or any treatment that you need |  | Did the doctor sufficiently explain your health problems and the necessary treatment so that you can understand them? |  | 9 |
| 10f | The amount of time your doctor spent with you today |  | Do you think the amount of time the doctor spent examining you was appropriate? |  | 8 |
| 10g | The doctor’s patience with your questions or worries |  | Did the doctor care about your symptoms and concerns, and offer helpful advice? |  | 8 |
| 10h | The doctor’s caring and concern for you |  | Do you think the doctor cared about, not just your medical condition, but also you as a person (your feelings and values, work and family situation)? |  | 9 |
| 11 | After seeing the doctor today do you feel… |  |  |  |  |
| 11a | able to understand your problem(s) or illness? |  |  |  | 8 |
| 11b | able to cope with your problem(s) or illness? |  | Combined |  | 7 |
| 11c | able to keep yourself healthy? |  |  |  | 8 |
| 12 | Gender |  |  |  |  |
| 13 | Age |  |  |  |  |
| 14 | Any long-standing illness, disability or infirmity |  |  |  |  |
| 15 | Ethnicity |  | Removed |  | 4 |
| 16 | Accommodation |  | Removed |  | 7 |
| 17 | Employment |  | Reworded |  |  |
| 18 | General comment |  |  |  |  |
