# Supplementary material for: Development of the Japanese version of the general practice assessment questionnaire: measurement of patient experience and testing of data quality
Source: BMC Fam Pract. 2018 Nov 28;19:181. doi: 10.1186/s12875-018-0873-8 (PMC6264598; doi:10.1186/s12875-018-0873-8)
Supplement: Supplementary file 2 — Contents of GPAQ-J. Question items – Japanese. (DOCX 15 kb) [file 12875_2018_873_MOESM2_ESM.docx]

| Additional file 2. Contents of GPAQ-J | |
| --- | --- |
| No. | Contents (in Japanese) |
| 1 | 過去1年間で、当院をおよそ何回受診されましたか？ |
| 2 | 当院の受付の対応について、どう思いますか？ |
| 3a | 現在の診療時間（曜日や時間帯）について、どう思いますか？ |
| 3b | 現在の診療時間に加えて、診療を希望する時間・曜日はありますか？ |
| 4a | 当院に着いてから診察までの待ち時間は通常どのくらいですか？ |
| 4b | この状況について、どう思いますか？ |
| 5a | 電話のつながり具合(呼び出し時間の長さなど)は、いかがでしたか？ |
| 5b | 質問や医学的な助言が欲しい時、医師と話をすることはできましたか？ |
| 5c | 質問や医学的な助言が欲しい時、医師または看護師の電話対応はいかがでしたか？ |
| 6 | 当院でいつも診てもらっている医師について質問します。 |
| 6a | あなたが当院で診察をうけようと思った時、その医師にどのくらいの頻度で、診てもらっていますか？ |
| 6b | この状況について、どう思いますか？ |
| 7 | 本日の診察について下記の質問にお答えください。 |
| 7a | 医師は、あなたの症状や心配事について十分に尋ねましたか？ |
| 7b | 医師は、あなたが話したかったことを十分に聞いてくれましたか？ |
| 7c | 医師は、身体診察をする際に（プライバシーや痛みなどに）十分に配慮していましたか？ |
| 7d | 医師は、治療の方針を決定する際にあなたと十分に話し合ってくれましたか？ |
| 7e | 医師は、問題点や必要な治療についてあなたが理解できるように十分に説明してくれましたか？ |
| 7f | 本日の診察時間の長さは適切であったと思いますか？ |
| 7g | 医師は、あなたの質問や心配事について親身になって相談に乗ってくれましたか？ |
| 7h | 医師は、あなたの病気だけでなくあなた自身（気持ちや価値観、職場や家族の状況）についても関心を持って診てくれたと思いますか？ |
| 8 | 診察前と診察後を比較して、下記の質問にお答えください。 |
| 8a | 診察前より、自分の健康問題や病気についての理解が深まりましたか？ |
| 8b | 診察前より、自分自身の健康管理に気を配ろうと思いましたか？ |
| 9 | 性別 |
| 10 | 年齢 |
| 11 | 職業 |
| 12 | あなたの健康について長期にわたる問題（病気や障がいなど）は、ありますか？ |
| 13 | 他に何かご意見がありましたら、こちらにお書きください。 当院の診療で、特に良いと思うところはありますか？ 当院の診療で、改善して欲しいと思うところはありますか？ 他にもご意見があれば、ご記入お願いします。 |
