# Supplementary material for: Development of the Japanese version of the general practice assessment questionnaire: measurement of patient experience and testing of data quality
Source: BMC Fam Pract. 2018 Nov 28;19:181. doi: 10.1186/s12875-018-0873-8 (PMC6264598; doi:10.1186/s12875-018-0873-8)
Supplement: Supplementary file 3 — Distribution of each item of GPAQ-J. Item descriptive statistics. (DOCX 17 kb) [file 12875_2018_873_MOESM3_ESM.docx]

| Additional file 3. Distribution of each item of GPAQ-J | | | | | | | | |
| --- | --- | --- | --- | --- | --- | --- | --- | --- |
| No. | Item | Distribution (%) | | | | | |  |
|  |  | Very poor | Poor | Fair | Good | Very good | Excellent | Does not apply |
| 2 | Receptionists | 1.6 | 0.8 | 7.6 | 22.5 | 46.5 | 21 | NA |
| 3a | Availability of surgery (any GP) | 0.4 | 6.9 | 30.2 | 29.0 | 21.2 | 12.2 | NA |
| 4b | Wating time at surgery | 1.2 | 19.8 | 40 | 18.6 | 12.4 | 7.9 | NA |
| 5a | Quality of phone call (length of time, etc.) | 0.4 | 0 | 11.8 | 15.9 | 37.7 | 12.7 | 21.4 |
| 5b | Availability of doctors on the phone | 0.4 | 0.9 | 9.9 | 12.6 | 34.1 | 16.6 | 25.6 |
| 5c | Doctor's or nurse’s phone support | 0.5 | 0.5 | 8.1 | 12.8 | 30.8 | 12.8 | 34.6 |
| 6b | Continuity of care | 38.5 | 26.3 | 21.5 | 6.8 | 5.4 | 1.5 | NA |
| 7a | GP questioning | 0 | 0.5 | 2.7 | 16.9 | 49.8 | 30.1 | NA |
| 7b | GP attention | 0 | 0.5 | 0.9 | 14.7 | 48.4 | 35.5 | NA |
| 7c | GP putting you at ease | 0 | 0 | 2.9 | 19.8 | 43.0 | 34.3 | NA |
| 7d | GP involving you in decisions | 0 | 0 | 1.9 | 14.9 | 49.8 | 33.5 | NA |
| 7e | GP explanations | 0 | 0 | 2.2 | 15.6 | 47.3 | 34.8 | NA |
| 7f | GP spending time with you | 0 | 2.3 | 8.1 | 17.6 | 37.4 | 34.7 | NA |
| 7g | GP patience | 0 | 0.5 | 1.8 | 14.2 | 43.1 | 40.4 | NA |
| 7h | GP caring and concern | 0 | 1.4 | 11.0 | 18.2 | 38.3 | 31.1 | NA |
|  |  | 1 time | 2-6 times | 7-12 times | 13 times or more | Don’t know/Don’t remember |  |  |
| 1 | Frequency of seeing a doctor during the previous 12 months | 0.4 | 24.5 | 50.2 | 24.9 | 0 |  |  |
|  |  | Currently good | Early morning | During lunch | Evening  (until 21:00) | Saturday | Sunday /holidays | Other |
| 3b | Do you have any preferences for times in addition to current office hours? | 65.9 | 1.2 | 1.6 | 6.0 | 10.7 | 6 | 3.2 |
|  |  | Less than  15 minutes | Between  15 and 30 minutes | Between  30 and 60 minutes | Between  1 and 2 hours | More than  2 hours |  |  |
| 4a | Waiting time to see a doctor | 12.6 | 38.2 | 36.6 | 11.0 | 1.6 |  |  |
|  |  | Always | Almost always | A lot of  the time | Some of  the time | Almost never | Never |  |
| 6a | Frequency you can see your own doctors | 38.5 | 26.3 | 21.5 | 6.8 | 5.4 | 1.5 |  |
|  |  | Much more than before the visit | A little more than before the visit | The same as before  the visit | Less than before  the visit | Does not apply |  |  |
| 8a | Able to understand your problem | 0 | 32.3 | 45.0 | 22.7 | 0 |  |  |
| 8b | Able to keep yourself healthy | 0 | 23.7 | 47.0 | 29.2 | 0 |  |  |
|  |  | Very dissatisfied | Somewhat dissatisfied | Neutral | Somewhat satisfied | Very satisfied |  |  |
|  | Overall satisfaction | 1.9 | 3.8 | 6.7 | 46.2 | 41.4 |  |  |
| GPAQ-J: Japanese version of General Practice Assessment Questionnaire | | | | | | | | |
| GP: General Practitioner | | | | | | | | |
| NA: Not applicable | | | | | | | | |
