# Supplementary material for: Development of the Japanese version of the general practice assessment questionnaire: measurement of patient experience and testing of data quality
Source: BMC Fam Pract. 2018 Nov 28;19:181. doi: 10.1186/s12875-018-0873-8 (PMC6264598; doi:10.1186/s12875-018-0873-8)
Supplement: Supplementary file 4 — Distribution of each item of JPCAT. Item descriptive statistics. (DOCX 15 kb) [file 12875_2018_873_MOESM4_ESM.docx]

| Additional file 4. Distribution of each item of JPCAT | | | | | | |
| --- | --- | --- | --- | --- | --- | --- |
| Scale | No. | Distribution (%) | | | | |
|  |  | Definitely not | Probably not | Not sure | Probably | Definitely |
| First contact (Access) | B1 | 1.4 | 7.6 | 13.8 | 45.2 | 31.9 |
|  | B2 | 9.4 | 29.2 | 28.2 | 25.2 | 7.9 |
|  | B3 | 9.7 | 32.3 | 31.3 | 24.6 | 2.1 |
| Longitudinality | C1 | 0.5 | 0 | 3.8 | 36.0 | 59.7 |
|  | C2 | 0.5 | 0 | 3.3 | 29.7 | 66.5 |
|  | C3 | 0.5 | 1.0 | 15.3 | 40.7 | 42.6 |
|  | C4 | 1.0 | 1.0 | 17.8 | 32.7 | 47.6 |
|  | C5 | 0 | 0.5 | 10.0 | 31.1 | 58.4 |
| Coordination | D1 | Experience of specialist consultation (YES, 71.7; No, 28.3) | | | | |
|  | D2 | 9.5 | 2.7 | 14.3 | 13.6 | 59.9 |
|  | D3 | 5.6 | 3.5 | 11.8 | 18.1 | 61.1 |
|  | D4 | 7.6 | 0.7 | 10.4 | 13.2 | 68.1 |
|  | D5 | 8.5 | 0.7 | 5.7 | 10.6 | 74.5 |
|  | D6 | 2.7 | 2.7 | 7.5 | 15.1 | 71.9 |
| Comprehensiveness (services available) | E1 | 1.0 | 3.5 | 16.3 | 41.1 | 38.1 |
|  | E2 | 1.0 | 1.5 | 7.4 | 40.7 | 49.5 |
|  | E3 | 0.5 | 1.5 | 9.3 | 37.7 | 51.0 |
|  | E4 | 1.1 | 2.2 | 21.5 | 39.2 | 35.9 |
|  | E5 | 1.6 | 2.6 | 20.8 | 39.6 | 35.4 |
| Comprehensiveness (services provided) | F1 | 8.6 | 8.1 | 15.2 | 27.3 | 40.9 |
|  | F2 | 15.7 | 13.1 | 24.1 | 17.8 | 29.3 |
|  | F3 | 21.4 | 17.1 | 28.9 | 17.1 | 15.5 |
|  | F4 | 25.4 | 18.0 | 32.3 | 13.8 | 10.6 |
|  | F5 | 13.2 | 11.6 | 22.2 | 31.2 | 21.7 |
| Community orientation | G1 | 1.0 | 0.5 | 6.1 | 16.5 | 75.8 |
|  | G2 | 0.5 | 0.5 | 7.3 | 28.3 | 63.4 |
|  | G3 | 0.5 | 0 | 7.4 | 39.4 | 52.7 |
|  | G4 | 1.7 | 0.6 | 30.2 | 44.7 | 22.9 |
|  | G5 | 1.7 | 0 | 24.0 | 48.6 | 25.7 |
|  | Mean (SD) | Very dissatisfied | Somewhat dissatisfied | Neutral | Somewhat satisfied | Very satisfied |
| Overall satisfaction | 4.43 (0.61) | 0 | 1.5 | 2 | 48.5 | 48.0 |
| JPCAT: Japanese version of the Primary Care Assessment Tool | | | | | | |
