# Supplementary material for: Development of the Japanese version of the general practice assessment questionnaire: measurement of patient experience and testing of data quality
Source: BMC Fam Pract. 2018 Nov 28;19:181. doi: 10.1186/s12875-018-0873-8 (PMC6264598; doi:10.1186/s12875-018-0873-8)
Supplement: Supplementary file 5 — Inter-scale correlations matrix. Reliability coefficients and inter-scale correlations. (DOCX 14 kb) [file 12875_2018_873_MOESM5_ESM.docx]

| Additional file 5. Inter-scale correlations matrix | | | | |  |
| --- | --- | --- | --- | --- | --- |
| Scale | Receptionists | Access | Continuity of care | Communication | Enablement |
| Receptionists | (-) | 0.5395 | 0.2926 | 0.3985 | 0.2675 |
| Access | 0.5395 | (0.79) | 0.4473 | 0.5083 | 0.3716 |
| Continuity of care | 0.2926 | 0.4473 | (-) | 0.361 | 0.2893 |
| Communication | 0.3985 | 0.5083 | 0.361 | (0.92) | 0.4083 |
| Enablement | 0.2675 | 0.3716 | 0.2893 | 0.4083 | (0.83) |
| Scale internal consistency reliability (Cronbach’s alpha coefficient) is described in the diagonal. | | | | | |
